# Quality and Consumer Acceptance of Chia Seed as an Egg Substitute in Brownies

**DOI:** 10.3390/foods14050882

**Published:** 2025-03-05

**Authors:** Laura Vu, Julie Kim, Moonkyu Margaret Choi, Jamie Kubota, Xi Feng

**Affiliations:** Department of Nutrition, Food Science, and Packaging, San José State University, San José, CA 95192, USA; laura.vu@sjsu.edu (L.V.); julie.kim@sjsu.edu (J.K.); moonkyu.choi@sjsu.edu (M.M.C.); jamie.kubota@sjsu.edu (J.K.)

**Keywords:** chia seeds, egg replacement, sensory quality, plant-based alternative

## Abstract

Chia seeds have emerged as a promising natural substitute for eggs in various baked products due to their unique gelling properties and ability to bind ingredients. Their gelling abilities closely mimic the moisture-retention functions of eggs in baked goods. The growing interest in plant-based alternatives creates a larger market for more sustainable foods. However, negative sensory attributes are found in baked goods with high chia seed content. The objective of this research was to explore the acceptance of chia gel as an egg replacer in brownies by documenting changes in product quality and chia functionality as an egg substitute. Brownies were made using Ghirardelli brownie mix, with two applied treatments containing chia gel, replacing 50 and 100 percent eggs (*w*/*w*). A sensory evaluation was performed with 120 participants to document their levels of acceptance of five attributes with a five-point hedonic scale: appearance, color, texture, consumer overall opinion, and purchase willingness. There were no significant differences between the 50% (*w*/*w*) substitution and control (*p* > 0.05). A 100% (*w*/*w*) substitution showed low acceptance for each attribute except aroma (*p* < 0.05). Flavor and taste were found to be leading determinants of overall opinion and purchase willingness (*p* < 0.05). These results highlighted the potential for chia seeds to be a viable alternative when replacing up to half of the egg content in brownies, while still maintaining sensory quality and satisfaction. Future research will explore the rheological properties of chia seed gels and their interaction with macro-/micro molecules in different food systems.

## 1. Introduction

Following the growing market for plant-based alternatives, finding an appropriate substitution for animal products has become imperative. One example is the substitution of eggs in food recipes, particularly in baked goods. For individuals with egg allergies, dietary restrictions, or those opting for plant-based diets, the search for a suitable egg replacement is essential [1]. However, current egg replacements have various sensory issues.

In recent years, chia seeds have emerged as a promising natural substitute for eggs in various baked products due to their unique gelling properties and ability to bind ingredients [2]. The hydrophilic nature of chia seeds allows their outer layer to absorb water and form a polysaccharide gel layer, or mucilage [3]. This gelling property closely mimics the binding and moisture-retention functions of eggs in baked goods [4]. However, excessive moisture binding can lead to microbial growth during storage as well as a negative consuming experience.

Chia is composed of 19–23% (*w*/*w*) protein and its digestibility is comparable to other sources of protein, such as corn, rice, and wheat [5,6]. Chia prepared as a protein, combined with other important food elements, expressed sufficiency to help prevent child malnutrition during infant feeding [7]. Moreover, the soluble fibers in chia are higher than those of quinoa, flaxseed, and amaranth at 30–34 g/100 g, meeting the daily intake for adults, and the soluble fibers in chia contribute to the texture and mouthfeel of baked products, enhancing their overall quality [7,8,9]. Additionally, chia seeds exhibit antioxidant properties that may help extend the shelf life of baked goods by reducing lipid oxidation [10].

However, some challenges were presented in studies regarding the sensory attributes of cakes containing chia mucilage. It affects the physical properties of cake, such as viscous and elastic behaviors, as well as lower volumes [11]. In terms of textural properties, chia showed low resilience values, which can affect the durability of the baked good, making it prone to falling apart easily and possibly making it look unappealing as a pastry [7]. The batter density increased with the chia seed addition, showing significant reduction in cake volume and providing denser cake crumbs. A higher value of chia was correlated to a lack of symmetry, darker crumbs, and greenness, likely due to the chia seed coating in the cake [12,13]. As a result, there was reportedly a lower sensory preference for the color, taste and texture when chia mucilage was added to replace the egg content [13].

The functional properties of chia mucilage express feasibility for food applications. However, negative sensory attributes found in baked goods with high chia content address the interest in improving chia processing for better functionality and quality as an egg replacement. The objectives of this study were to (1) evaluate consumer sensory preferences and changes in the quality of brownies made with chia mucilage compared to standard brownie formulations made with eggs; (2) elucidate the components responsible for the quality deterioration. The results of this study should provide a better understanding of the potential advantages or disadvantages of chia seeds as an egg replacement, and the conclusions can be extrapolated to other bakery products. Also, the study will provide a theorical foundation for nutrition education to decrease the barrier for consumers to adopt chia seeds as an egg replacement.

## 2. Materials and Methods

### 2.1. Participants

The study received approval from the Institutional Review Board (IRB) of San José State University (SJSU). Participants (*n* = 120) were recruited using promotional fliers, department emails, and in-person recruitment. Interested individuals were directed to enroll themselves in the study via a provided link and QR code in the promotional flier. Participants provided informed consent before participating in the study. Consent was obtained in-person at the study site by the researchers. Participants were required to be San Jose State University students, faculty, or staff, and 18 years and older. Individuals with egg, soy, and gluten allergies were excluded from participation.

### 2.2. Sample Preparation

Ghirardelli Chocolate Supreme Premium Brownie Mix, chia seeds, eggs, vegetable oil, and 1-inch cupcake liners were procured from the local supermarket (San Jose, CA, USA). The syrup packet included in the brownie mix packaging was excluded from the brownie formulations. This was conducted to maintain the standardization of brownie recipes without inclusions and decrease variability among the sample.

Chia gel was obtained by grinding the chia seeds into a powder using a kitchen blender for 1 min and storing it in an airtight container at 0 °F until further use. Based on our preliminary test results, chia gel was made by combining 2 tablespoons of ground chia (30 g) with 5 tablespoons of water (74 g). This mixture was stirred then allowed to gel for 10 min in the refrigerator. While waiting for the mixture to gel, the dry mix was sifted into a bowl and the other wet ingredients were measured. Once the chia mixture had gelled, it was added to the wet ingredients. A hand mixer was used to mix the wet ingredients for 2 min at 180 rpm [8]. For the control, the brownies were made following the box instructions using a regular egg. For the 50% (*w*/*w*) brownie, half an egg and chia gel were used to substitute one whole egg (44 g); 23.5 g of the chia gel was used to replace 23.5 g of the egg. For the 100% (*w*/*w*) brownie, 44 g of the chia gel replaced 100% of the egg in the formulation. The brownies were baked in 1-inch cupcake liners for 12 min at 325 °F, then allowed to cool for half an hour at room temperature. After being cooled, the brownies were double wrapped in plastic wrap, zipped into an airtight bag, and placed in the freezer (−18 °C) until ready for sensory evaluation. The brownies were defrosted at room temperature for 1–2 h before serving.

### 2.3. Sensory Evaluation

The sensory evaluation of the brownies was conducted at San José State University in the span of 6 days to assess which sensory attributes were significant amongst the three brownie samples—the control, 50% (*w*/*w*) egg substitution, and 100% (*w*/*w*) egg substitution. Brownies were baked on-site in the nutrition department teaching kitchen laboratory, and the sensory evaluation was conducted in a department classroom set up with separate stations for participants.

A demographic survey was given to each participant before the sensory tests. The brownie samples were each coded with a 3-digit code. To reduce bias, the brownie samples were placed at random in front of participants. Room temperature water was provided as a palate cleanser in between each brownie tasting to minimize contamination between samples. The sensory evaluation of the brownie samples was conducted using a 5-point hedonic scale, in which the value 1 indicated “extremely dislike”, and 5 indicated “extremely like”. Consumer preferences were based on the quality attributes of aroma, flavor, taste, appearance, crumb texture, opinion, and purchase willingness. No identity information was collected.

### 2.4. Chemical Analysis

Compositional analysis was performed using AOAC methods [14]. Moisture content was determined by weight loss after 12 h at 105 °C in a drying oven. Water activity was measured with a water activity meter (AquaLab, Corona, CA, USA). Protein content was determined by measuring nitrogen content and applying a conversion factor of 6.25 using combustion methods (LECO FP828 Analyzer, St. Joseph, MI, USA) [15]. Fat content was measured using acid-hydrolysis methods with petroleum ether and ethyl ether (1:1, *v*/*v*) solvent extraction [16]. Carbohydrate content was calculated by 100% − (Moisture% + Protein% + Fat%). Calorie values were calculated by macronutrients (carbohydrates, protein, and fat); carbohydrates: 4 calories per gram, protein: 4 calories per gram, fat: 9 calories per gram [17]. For each of these tests, three replications were conducted.

### 2.5. Statistical Analysis

The number of participants and percentages were recorded for gender and ethnicity; mean and standard deviation were additionally recorded for age. The statistical analysis software XLSTAT 2024 was used to apply a one-way analysis of variance (ANOVA) to measure the differences among the sample means by Tukey’s multiple comparison method. The Pearson correlation tests were employed to measure the correlation among appearance, crust color, crumb color, texture, consumer opinion, purchase willingness, moisture contents, water activities, protein, and fat contents, and the significance level was set at *p* < 0.05. Additionally, a PCA was conducted to examine any patterns and the magnitude of the relationship among variables.

## 3. Results

### 3.1. Participant Demographics

As participant recruitment occurred within a college setting, the average age of the participants who took part in the study was approximately 25 years old (Table 1). A larger percentage of participants were female, at 67.6%, while the male population represented a smaller percentage at 30.6%; 0.9% of participants identified as non-binary or undisclosed (Table 1). This is a common trend in many college populations, where there is a larger proportion of female students than male. The distribution of the ethnic demographics was skewed heavily towards Asian, at 53.2%, followed by White at 16.2%, Other at 12.6%, and Two or More at 10.8%. The Black, Native American/Pacific Islander, and N/A populations made up 7.2% of the participants altogether. The ethnic demographics reflect the local population and the university’s demographic makeup.

### 3.2. Sensory Analysis

**Figure 1:**
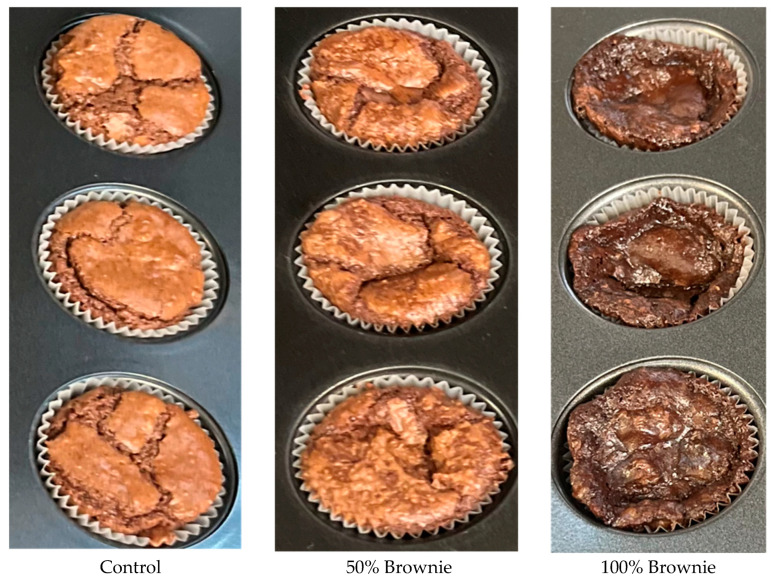
Appearance of brownies.

The brownies made with 100% egg substitution (*w*/*w*) exhibited a notably darker color and a visibly collapsed appearance compared to the control group (Figure 1). Although the color of the crust and crumb both showcased a lighter brown color, the 50% brownie (*w*/*w*) appeared to have a slightly puffed-up appearance, with a more textured surface. On the other hand, the 100% substitution (*w*/*w*) looked deflated and deformed, with an irregular surface area; it also failed to fill out the cupcake mold like the control and the 50% brownie (*w*/*w*). The crumb of the 100% brownie (*w*/*w*) also evidently lacked structural integrity due to the sticky, airy pockets, and the fragile crust.

The quality of the brownies was determined by aroma, taste, flavor, appearance, and texture (Table 2). The addition of 100% and 50% egg substitution (*w*/*w*) showed no significant difference in aroma compared to control. However, 100% substitution of egg (*w*/*w*) resulted in significantly low sensory preference across each measured attribute. It was the least preferable, with overall opinion and purchase values lacking in comparison to the 50% and control groups. This suggests that a complete replacement of egg with chia gel negatively impacts the sensory experience of the brownie and participants’ willingness to purchase.

Sensory data found that 50% egg substitution (*w*/*w*) was perceived to have the highest acceptability across each quality attribute based on the mean value. Crust and crumb texture notably scored significantly higher in 50% egg substitution (*w*/*w*) than the control, resulting in a more structured interior that was preferred by panelists. This indicates that the sensory attributes of brownies with 50% egg substitution (*w*/*w*) were well accepted by consumers. Despite this, there was no significant difference between this level of substitution and the control group. This suggests that replacing up to half of the eggs with chia gel in brownies can create a product with high similarity to the sensory attributes of a standard brownie.

### 3.3. Chemical Analysis

It was observed that there was a significant difference between the brownies with 50% egg substitution (*w*/*w*) and the control group in moisture content, but there was no significant difference between the control and 100% egg substitution (Table 3). The 100% brownie (*w*/*w*) had the highest moisture content, which can be attributed to the additional water added when incorporating the chia mixture as the egg replacement. Thus, the extra water from the chia mucilage may have contributed to the additional moisture content in the brownie. For water activity, there were significant differences among all three samples. The 100% egg substitution (*w*/*w*) resulted in a significantly higher value of water activity, demonstrating the risk of having abundant free-bound water available for microbial growth and resulting in a shorter shelf life. Natural preservatives, such as sorbic acids, can be added to minimize mold growth. The 50% egg substitution (*w*/*w*) had the lowest water activity, of less than 0.5; this brownie is safe to be considered as dry food and will be stable without refrigeration for an extended period of time. In addition to exhibiting the lowest moisture content at 9.987%, brownies with 50% egg substitution (*w*/*w*) would have the lowest chance for spoilage or bacteria to grow rapidly. However, considering that moisture content is a critical factor influencing the texture of brownies, this relationship should be carefully evaluated in the data analysis to understand its implications on the overall product quality.

Protein content also demonstrated significant differences among the three samples. Notably, the control measured the highest protein content due to the presence of eggs used as thickeners and emulsifiers. Eggs are a great source of complete protein, with a high protein digestibility-corrected amino acid score (PDCAAS), contributing to a higher percentage of protein in brownie samples. Chia seeds, however, are not a protein-rich ingredient. Although they contain more protein than corn, rice, and wheat, it is insufficient to replace the same amount of protein eggs could contribute. Thus, 100% egg substitution (*w*/*w*) had a significantly lower protein content than 50% egg substitution (*w*/*w*) and the control. Fat content, on the other hand, did not demonstrate any significant differences among the three samples. Chia seeds are characterized by their high content of polyunsaturated omega-3 fatty acids, while eggs contain monounsaturated and saturated fat, both contributing to the high fat content in each sample. The 100% egg substitution (*w*/*w*) showed the lowest fat percentage, whereas 50% egg substitution (*w*/*w*) had the highest. This is likely due to the diverse dietary fats available in the 50% egg substitution (*w*/*w*) sample, with the presence of both chia seeds and eggs. Significant differences were found in carbohydrate contents and calories among the three samples. The 50% egg substitution had the highest carbohydrate content and calorie value, which was due to its lower moisture content.

### 3.4. Correlation Analysis

Pearson’s correlation test was used to examine the relationships among aroma, taste, flavor, appearance, crust color and texture, crumb color and texture, overall opinion, purchase willingness, and chemical parameters (Table 4). The aroma had a significant correlation with all variables except water activity, moisture, protein, and fat % (g/g). It especially had a strong positive correlation with flavor, because the volatiles detected by the olfactory system play an essential role in detecting the overall flavor of a food product. The more aromatic the brownie was, the more likely panelists identified a liking of the flavor. However, aroma had the weakest correlation to purchase willingness, followed by color and texture, suggesting its minimal influence on a consumer’s intention to purchase.

While purchase willingness found high correlations among variables, it was especially heavily influenced by overall opinion, flavor, and taste. The significant relation between flavor and opinion suggests that the participants’ overall opinions of the brownies were heavily influenced by their perceptions of the flavor. In this interpretation, the more flavorful the sample was, the more positive the overall opinion. Similarly, the brownie would be preferred by the consumer if the brownie had a favorable taste in sweetness, saltiness, and bitterness categories. The relationship between opinion and purchase indicates that participants who had positive opinions of the brownie expressed more purchase willingness or higher intention to buy.

Moisture % (g/g) and water activity observed a strong negative correlation with all variables. Moisture % (g/g) especially had a significant correlation with crumb texture; the higher the moisture content of the brownie, the less brittle the crumbs were perceived to be by consumers. Crumb texture had a stronger influence on the moisture content than the crust texture, illustrating the importance of maintaining moisture inside the brownie to achieve a fudgy texture. Water activity had the strongest negative correlation with purchase willingness. Consumers are more likely to purchase a brownie with lower water activity, likely due to the extended shelf life. The potential of quick spoilage of high moisture/water activity food outweighs the immediate sensory experience. A food with a lower moisture and water acidity value is perceived as stable during storage.

### 3.5. Principal Component Analysis

**Figure 2:**
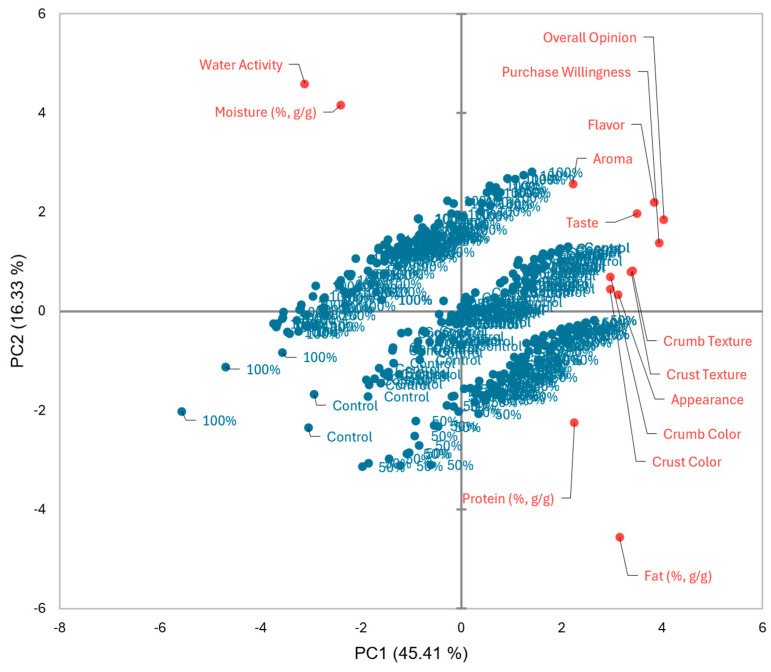
Principal component analysis of brownie samples.

Principal component analysis (PCA) was conducted to illustrate the contribution of each attribute to the samples (Figure 2). The first component (PC1) demonstrates 45.41%, while the second component (PC2) demonstrates 16.33% of the variation, with a total of 61.74%. The 100% egg substitution (*w*/*w*) was primarily affected by the aroma, moisture and water activity. The control group and 50% egg substitution (*w*/*w*) were associated with appearance, color of crust and crumb, texture of crust and crumb, taste, flavor, protein and fat content. This is due to the off-color 100% egg substitution (*w*/*w*) produced, resulting in panelists focusing on the other two samples with similar color and texture.

## 4. Discussion

The results showed that a 100% egg substitution (*w*/*w*) negatively impacted sensory preference across all attributes except aroma, suggesting that complete replacement may not be ideal for maintaining product quality. Chia seed can retain a significant amount of water, as shown in Table 3. It may decrease the dryness of brownies but enhance sweet perception among consumers [18]. The gel-like texture of chia seeds can make the brownies more chewy, gummy or grainy. A noticeable nutty flavor was also reported in chia seed (*Salvia hispanica* L.) and extra virgin olive oil additions to salty crackers [19]. However, a 50% egg substitution (*w*/*w*) was well-received and closely resembled the control group in sensory attributes, indicating that partial egg substitution with chia gel could be a viable option for maintaining brownie quality. Additionally, the study highlighted the importance of flavor and taste in shaping consumer opinions and purchase decisions. This suggests that while chia gel can effectively replace up to 50% of the egg (*w*/*w*), optimizing flavor and taste is crucial to enhancing consumer acceptance and willingness to purchase. This provides insight into the potential of chia gel as an egg substitute in brownies, emphasizing the need for a balanced formulation to ensure both product quality and consumer acceptance.

Similarly, studies using chia in their formulations to replace egg, oil, or flour at percentages over 50% resulted in lower sensory attributes and decreased quality. Borneo et al. [13] were able to substitute egg or oil with chia gel in cakes for up to 25% without decreasing sensory acceptance. Researchers found no significant difference between the control cake and those made with 25% oil or 25% chia substitution (*w*/*w*). Aljobair found that eggs were replaceable by up to 25% in sponge cake, with equal acceptability to control [12]. Similar results were found in bread. A study on the fortification of cheese bread with chia (5–10% *w*/*w*) found no significant change in the sensory attributes [20]. Another study on the fortification of bread with chia (2–6% *w*/*w*) similarly found no statistically significant difference in overall sensory attributes among all bread samples [21].

Consumer acceptance of chia-based baked products has been positive, with many consumers appreciating the added nutritional benefits and sensory qualities provided by chia seeds [22]. Chia seeds are rich in dietary fibers, omega-3 fatty acids, and phenolic compounds, which have antioxidant or anti-inflammatory effects. They can also improve glucose metabolism and dyslipidemia [23]. Chia seeds have up to 8.8% polyphenolic compounds [6]. In addition, they have isoflavones (e.g., daidzein, glycitin, genistein, etc.) and their derivatives (e.g., catechin, epicatechin, etc.) [24]. With a low amount of carotenoids, tannins, and phytates, chia seeds display health promotion properties and potential [25,26]. However, further research is needed to optimize chia seed incorporation levels and baking conditions to achieve the desired texture, taste, and shelf stability of chia-enriched baked goods [27]. Though available data support the potential role of chia as a functional ingredient and substitute in baked goods, lower sensory preference presents a challenge to the total substitution of chia as an egg substitute. The negative sensory attributes found in baked goods with high chia content address the interest in improving chia processing for better functionality as an egg replacement, specifically in structure. There were some limitations in this study; this study exclusively used the Ghirardelli Chocolate Supreme Brownie Mix, which may not be representative of all the brownie mixes on the market. The study recruited 120 participants, all from San José State University, which limits generalizability. Future studies can recruit broader populations. Also, implementing double-blinding could possibly further minimize potential bias in sensory evaluations, as consumers were able to detect the appearance differences among the samples.

## 5. Conclusions

The results of sensory evaluation provide insight into the influential factors of consumer preferences and purchase intentions regarding brownies. While aroma had minimal impact on purchase willingness, taste, and flavor emerged as critical determinants of opinion. These results highlight the potential for chia seeds to be a viable alternative when replacing up to half of the egg content in brownies, while still maintaining sensory quality and satisfaction in the areas of aroma, taste, flavor, appearance, color and texture. The nutrient benefits can also improve consumer acceptance with chia seeds. For baked goods that have a darker color for their end product, chia substitution may be optimal as it can help mask the color change it may cause, as it did with the 100% brownie. Due to chia seeds mainly being produced in South America (e.g., Argentina, Paraguay, Bolivia), the scalability may be limited. The cost of chia seeds is higher than traditional ingredients (e.g., egg, etc.). However, given the increasing consumer demands for health-conscious and functional foods, chia seed-added products could be competitive in the market. Future research can focus on characterizing the structure of chia gel to determine its efficacy in replicating egg structures in baked goods, as well as examining the relationship between the shelf life of chia seed brownies with different water activity levels.

## Figures and Tables

**Table 1:** Participant demographics (*n* = 120).

Sample Characteristics	n	%	(m ± sd)
Age			25.28 ± 8.25
Gender			
Female	73	67.6	
Male	34	30.6	
Non-Binary	1	0.9	
N/A	1	0.9	
Ethnicity			
Asian	59	53.2	
White	17	16.2	
Native American/Pacific Islander	3	2.7	
Black	1	0.9	
Other	14	12.6	
Two or More	12	10.8	
N/A	4	3.6	

**Table 2:** Sensory attributes of brownie sample.

	Control	50% Chia Seeds	100% Chia Seeds	SEM	*p*-Value
Aroma	4.05 ^a^	4.15 ^a^	4.02 ^a^	0.04	0.426
Taste	4.11 ^a^	4.31 ^a^	3.68 ^b^	0.05	<0.0001
Flavor	4.12 ^a^	4.28 ^a^	3.67 ^b^	0.04	<0.0001
Appearance	4.02 ^a^	3.90 ^a^	3.08 ^b^	0.05	<0.0001
Crust Color	4.09 ^a^	3.93 ^a^	3.24 ^b^	0.05	<0.0001
Crumb Color	3.94 ^a^	4.03 ^a^	3.43 ^b^	0.04	<0.0001
Crust Texture	4.03 ^b^	4.36 ^a^	3.51 ^c^	0.05	<0.0001
Crumb Texture	3.89 ^b^	4.29 ^a^	3.40 ^c^	0.05	<0.0001
Overall Opinion	4.07 ^a^	4.31 ^a^	3.51 ^b^	0.05	<0.0001
Purchase Willingness	3.69 ^a^	3.98 ^a^	2.93 ^b^	0.05	<0.0001

^a,b,c^ Means with different letters within a row differ significantly (*p* < 0.05).

**Table 3:** Compositional analysis of brownie samples.

	Control	50% Chia Seeds	100% Chia Seeds	SEM	*p*-Value
Moisture (%)	12.37 ^a^	9.99 ^b^	12.40 ^a^	0.26	0.027
Water Activity	0.56 ^b^	0.48 ^c^	0.64 ^a^	0.01	0.0003
Protein (%)	4.44 ^a^	4.13 ^b^	3.75 ^c^	0.04	0.001
Fat (%)	12.31 ^a^	13.14 ^a^	11.30 ^a^	0.36	0.272
Carbohydrate (%)	70.89 ^b^	72.75 ^a^	72.56 ^a^	0.12	0.001
Calories (cal/100 g)	412.07 ^ab^	425.74 ^a^	406.88 ^b^	2.03	0.02

^a,b,c^ Means with different letters within a row differ significantly (*p* < 0.05).

**Table 4:** Pearson’s correlation among sensory attributes and chemical parameters of brownie samples.

	Aroma	Taste	Flavor	Appearance	Crust Color	Crumb Color	Crust Texture	Crumb Texture	Overall Opinion	Purchase Willingness	Moisture (%, g/g)	Water Activity	Protein (%, g/g)	Fat (%, g/g)
Aroma	**1**	**0.420**	**0.514**	**0.241**	**0.220**	**0.276**	**0.347**	**0.333**	**0.467**	**0.359**	−0.067	−0.066	0.020	0.065
Taste		**1**	**0.786**	**0.331**	**0.321**	**0.324**	**0.466**	**0.498**	**0.757**	**0.715**	**−0.213**	**−0.278**	**0.202**	**0.281**
Flavor			**1**	**0.414**	**0.401**	**0.436**	**0.546**	**0.559**	**0.828**	**0.752**	**−0.217**	**−0.293**	**0.226**	**0.297**
Appearance				**1**	**0.708**	**0.664**	**0.383**	**0.321**	**0.482**	**0.440**	**−0.165**	**−0.325**	**0.380**	**0.338**
Crust Color					**1**	**0.694**	**0.324**	**0.320**	**0.425**	**0.397**	**−0.133**	**−0.290**	**0.363**	**0.303**
Crumb Color						**1**	**0.373**	**0.328**	**0.413**	**0.407**	**−0.182**	**−0.272**	**0.244**	**0.278**
Crust Texture							**1**	**0.653**	**0.592**	**0.585**	**−0.286**	**−0.353**	**0.231**	**0.355**
Crumb Texture								**1**	**0.623**	**0.633**	**−0.303**	**−0.360**	**0.215**	**0.360**
Overall Opinion									**1**	**0.816**	**−0.263**	**−0.347**	**0.257**	**0.351**
Purchase Willingness										**1**	**−0.281**	**−0.378**	**0.291**	**0.383**
Moisture (%, g/g)											**1**	**0.871**	−0.069	**−0.842**
Water Activity												**1**	**−0.550**	**−0.998**
Protein (%, g/g)													**1**	**0.596**
Fat (%, g/g)														**1**

Values in bold are different from 0 with a significance level of alpha = 0.05.

## Data Availability

The raw data supporting the conclusions of this article will be made available by the authors on request.
